# Epigenetic alterations and advancement of treatment in peripheral T-cell lymphoma

**DOI:** 10.1186/s13148-020-00962-x

**Published:** 2020-11-07

**Authors:** Ping Zhang, Mingzhi Zhang

**Affiliations:** 1 Department of Oncology, The First Affiliated Hospital of Zhengzhou University, Zhengzhou City, 450052 Henan Province China; 2 Academy of Medical Sciences of Zhengzhou University, Zhengzhou City, 450052 Henan Province China

**Keywords:** Epigenetics, Pathogenesis, Peripheral T-cell lymphoma, Epigenetic drugs, Therapy progressions

## Abstract

Peripheral T-cell lymphoma (PTCL) is a rare and heterogeneous group of clinically aggressive diseases associated with poor prognosis. Except for ALK + anaplastic large-cell lymphoma (ALCL), most peripheral T-cell lymphomas are highly malignant and have an aggressive disease course and poor clinical outcomes, with a poor remission rate and frequent relapse after first-line treatment. Aberrant epigenetic alterations play an important role in the pathogenesis and development of specific types of peripheral T-cell lymphoma, including the regulation of the expression of genes and signal transduction. The most common epigenetic alterations are DNA methylation and histone modification. Histone modification alters the level of gene expression by regulating the acetylation status of lysine residues on the promoter surrounding histones, often leading to the silencing of tumour suppressor genes or the overexpression of proto-oncogenes in lymphoma. DNA methylation refers to CpG islands, generally leading to tumour suppressor gene transcriptional silencing. Genetic studies have also shown that some recurrent mutations in genes involved in the epigenetic machinery, including TET2, IDH2-R172, DNMT3A, RHOA, CD28, IDH2, TET2, MLL2, KMT2A, KDM6A, CREBBP, and EP300, have been observed in cases of PTCL. The aberrant expression of miRNAs has also gradually become a diagnostic biomarker. These provide a reasonable molecular mechanism for epigenetic modifying drugs in the treatment of PTCL. As epigenetic drugs implicated in lymphoma have been continually reported in recent years, many new ideas for the diagnosis, treatment, and prognosis of PTCL originate from epigenetics in recent years. Novel epigenetic-targeted drugs have shown good tolerance and therapeutic effects in the treatment of peripheral T-cell lymphoma as monotherapy or combination therapy. NCCN Clinical Practice Guidelines also recommended epigenetic drugs for PTCL subtypes as second-line therapy. Epigenetic mechanisms provide new directions and therapeutic strategies for the research and treatment of peripheral T-cell lymphoma. Therefore, this paper mainly reviews the epigenetic changes in the pathogenesis of peripheral T-cell lymphoma and the advancement of epigenetic-targeted drugs in the treatment of peripheral T-cell lymphoma (PTCL).

## Background

Peripheral T-cell lymphomas (PTCLs) originate from post-thymic, mature T-cells or NK/T-cells and are a group of non-Hodgkin lymphomas (NHL) with highly heterogeneous morphologic changes. Classification of mature T and NK cell lymphomas and lymphoproliferative disorders is more than 27 subtypes, according to the 2016 revision of the WHO classification of lymphoid neoplasms [1, 2]. PTCLs consist mainly of 8 subtypes, including peripheral T-cell lymphoma not otherwise specified (PTCL, NOS), angioimmunoblastic T-cell lymphoma (AITL), ALK-positive anaplastic large-cell lymphoma (ALCL), ALK-negative ALCL, enteropathy-associated T-cell lymphoma (EATL), monomorphic epitheliotropic intestinal T-cell lymphoma (MEITL, also referred as type EATL-II), nodal peripheral T-cell lymphoma with TFH phenotype (PTCL-TFH), and follicular T-cell lymphoma (FTCL), according to NCCN Clinical Practice Guidelines in Oncology: T-Cell Lymphomas Version 1.2020 T-Cell Lymphomas [3]. Approximately 30–50% of lymphomas have not been further classified and are considered to be PTCL-NOS [4]. Of all cases of NHL in China, approximately 25–30% are PTCLs, which is significantly higher than that in European and American countries (10–15%) [5]. At present, the first-line treatment is still CHOP (cyclophosphamide + doxorubicin + vincristine + prednisolone) or a CHOP-like regimen (CHOEP). However, except for ALK + anaplastic large-cell lymphoma (ALCL), most peripheral T-cell lymphomas (PTCLs) are highly malignant and have an aggressive disease course and poor clinical outcomes, with poor remission rates and frequent relapse after first-line treatment [6]. Epigenetics refers to the phenomenon in which changes in gene expression occur without changes to the DNA sequence, and some epigenetic mechanisms of cell regulation include histone modification, DNA methylation, noncoding RNA effects, and chromatin reorganization. Abnormal activity of HDACs often affects genetic expression, causing the epigenetic silencing of tumour suppressor genes and oncogene activation. Moreover, genetic studies have shown that mutations in epigenetic modifying genes, including TET2, IDH2-R172, DNMT3A, RHOA, IDH2, TET2, MLL2, KMT2A, KDM6A, CREBBP, and EP300, have been observed in cases of PTCLs (Table 1) [7]. In particular, RHOA G17V [8], IDH2 and TET2 mutations were more likely to cause subtypes to manifest a T-follicular helper phenotype, as occurs with AITL and PTCL-NOS [9–11]. Histone modifier gene mutations were associated with disease progression in PTCL, sensitizing T-lymphoma cells to epigenetic drugs [12]. The effect of aberrant epigenetic modifier changes on multistep lymphomagenesis is thought to be essential for the pathogenesis of PTCL [13, 14]. However, such epigenetic changes are reversible and can be inhibited by drugs such as deacetylase inhibitors and EZH2 inhibitors to regulate the activity of histone-modifying enzymes and thus histone structure and gene expression, producing wide antitumour effects in peripheral T-cell lymphoma.

**Table 1:** The mutational landscape of PTCL

Mutational gene	Most frequent subtype	frequency	Frequent mutation in tumour type
TET2 [9, 10, 14, 116]	AITL, PTCL-NOS	42-89%, 28-48, 5%	MPN (~13%), (CMML) (~50%), MDS (25%), AML(~23%) [154]
DNMT3A [10, 14]	AITL, PTCL-NOS	25-33%, 27%	AMLs (20–30%), MDS (10–15%) [155]
IDH2	AITL [11] PTCL-NOS	20-45%, 7.5%^156^	AML(8–19%) [157] MDS (~5%) [158]
KMT2D/MLL2 [12, 58, 93]	All PTCL, AITL, PTCL-NOS	42%, 25%, 36%	DLBCL (35–85%) [159] FL (89%) [160]
KMT2C, SETD1B	PTCL, NOS [93, 156]	8.2%, 5.2%	Breast [161] cancer 8%MLL2+MLL3(16-20%) medulloblastoma [162, 163]
SETD2	EATL MEITL	32%, [83] 21/23 (91%) [84]	Renal cell carcinoma (13-30%) [164]

TET: ten eleven translocation protein, 2HG:2-hydroxyglutarate, IDH2: isocitrate dehydrogenase2, 5hmC: 5-hydroxymethylcytosine, EZH2: enhancer of zeste 2, MDS: myelodysplastic syndromes, AML: acute myeloid leukaemia

## Histone modification in the pathogenesis of peripheral T-cell lymphoma

### Histone acetylation

**Fig. 1:**
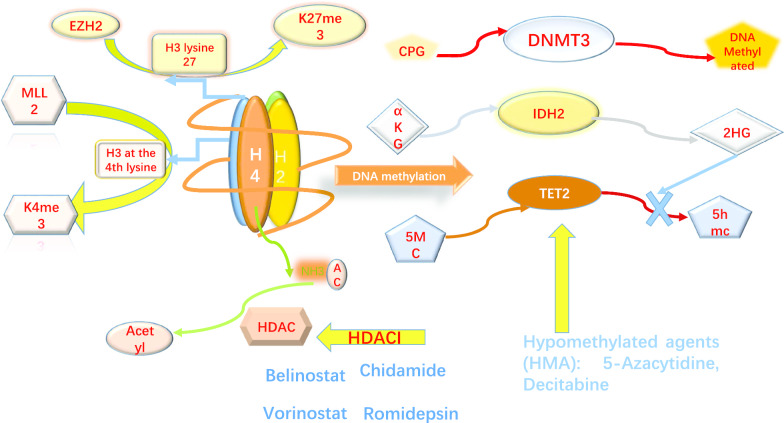
Epigenetics alteration and mechanism in PTCL. MLL2:MLL2 can activate the transcription of genes by methylating histone H3 at the 4th lysine (H3K4me).EZH2:EZH2 inhibits gene expression by catalysing trimethylation of lysine 27 of histone H3 (H3K27m3).HDAC : HATs catalyse the transfer of an acetyl group from acetyl- CoA to the NH2 group of lysine residues of histone H3 and histone H4 while HDACs remove it. DNMTs: DNMTs catalyse the transfer of methyl groups to cytosine nucleotides of CpG island DNA.TET2: encodes TET which converts 5-methylcytosine (5mC) to 5-hydroxymethylcytosine (5hmC). When TET2 is mutated, there is a pathogenic decrease in 5hmC leading to suppression of gene transcription.IDH2: Mutations of IDH2, such as gain-of-function R140 and R172 substitutions, lead to toxic 2-hydroxyglutarate (2HG) accumulate; which inhibits TET and decreases the levels of 5hmC. TET: ten eleven translocation protein; 2HG: 2-hydroxyglutarate; IDH2:isocitrate dehydrogenase 2; 5hmC: 5-hydroxymethylcytosine; EZH2: enhancer of zeste 2; K27me3: trimethylation at lysine 27 of histone 3 (H3K27me3); K4me3: trimethylation at lysine 4 of histone 3 (H3K4me3)

Histone modifications include histone acetylation, methylation, phosphorylation, and ubiquitination, and one of the most frequent aberrations is the acetylation of histones. Histone acetyltransferases (HATs) and histone deacetylases (HDACs) balance each other in the acetylation and deacetylation of histones and play an important role in chromosome structural modification and the regulation of gene expression [15, 16]. HATs catalyse the transfer of an acetyl group from acetyl-CoA to the NH2 group of lysine residues in proteins, while HDACs remove acetyl groups (Fig. 1). HDACs are often aberrantly expressed in peripheral-T-cell lymphoma and contribute to disease progression and poor prognosis; for example, HDAC6 overexpression in PTCL is associated with poor outcomes [17, 18]. As a study reported, low levels of HDAC7 and [19] HDAC1/2 activity are required for T-cell lymphoma development. Furthermore, the inactivation of HDAC3 alters the expression of the majority of genes at significantly lower levels, which affects cell cycle progression and functional T-cell transformation [20]. High expression of HDAC2 frequently occurs in PTCL patients. Huilai Zhang et al. enrolled 82 patients to systematically investigate the potential associations between HDAC or EZH2 expression and prognosis in PTCL subtypes. The results revealed that EZH2 and HDAC1/2 were frequently upregulated in patients with PTCL, and patients with higher EZH2 and HDAC2 expression usually exhibited a poorer survival rate [21]. HDACs, which are frequently dysregulated in cancer, represent the products of 18 genes and can be subdivided into 4 classes according to their homology with yeast HDACs, subcellular localization and enzymatic activity. Table 2 summarizes all classifications of histone deacetylase enzymes [22].

**Table 2:** Classification and functions of histone deacetylase enzymes

HDAC classification	Subtypes	Location	Function/role
Class I	HDAC1, HDAC2, HDAC3, HDAC8	Nucleus	Participate in the regulation of histone deacetylation modification
Class II	IIAHDACs (HDAC4, HDAC5, HDAC7, HDAC9)	Shuttle between cytoplasm and nucleus	In response to mitogenic signals [165]
	IIb-HDACs (HDAC6 HDAC10)	Cytoplasm	HDAC6 is reported to modulate the function of the non-histone protein HSP90 [166]
Class III [167]	Seven members of this family are SIRT1 through SIRT7	SIRTs are located in cellular compartments: nucleus, cytoplasm, and mitochondrion	Involved in cell ageing and energy metabolism processes [168–170]
Class IV	HDAC11	nucleus	Implicate in the regulation of interleukin-10 expression [171, 172]

**Fig. 2:**
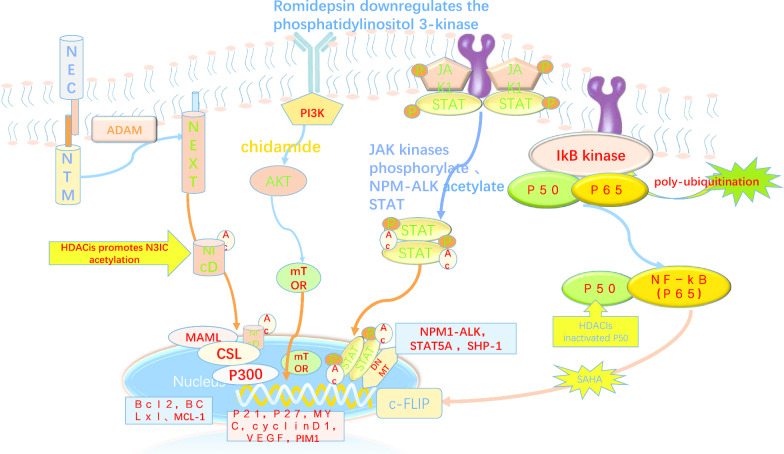
HDACis affect signalling pathways and oncogenes in PTCL. HDACI-mediated c-FLIP downregulation was related to NF-Κb members P50 inactivation via interrupting p50 interaction with c-FLIP. HDACIs inactivated P50 through inhibiting HDAC1.Romidepsin、chidamide downregulates (PI3K-AKT-mTOR) pathway by decreasing the phosphorylation of the p85 regulatory subunit of PI3K, which correlates with the observed decrease in the phosphorylation status of AKT. Histone acetyltransferase p300–mediated Stat3 acetylation on Lys685,STAT3 acetylation mediates the STAT3–DNMT1 interaction to regulate tumour suppressor gene promoter methylation. Acetylated STAT3 mediates epigenetic tumour suppressor gene (TSG) silencing, SHP-1. Notch3 intracellular domain (N3IC) is acetylated and deacetylated at lysines 1692 and 1731 by p300 and HDAC1, this modification reduces Notch3 protein stability. HDACi trichostatin (TSA) promotes N3IC acetylation ,leading to N3IC proteasomal degradation and downregulating N3IC-triggered signalling. downstream of the pathway pro-apoptotic and anti-apoptotic genes are affected

In addition to interfering with epigenetic chromatin modification, HDACs and HATs can also modify many oncoproteins and signalling pathway non-histone proteins, such as signal transduction and activator of transcription 3 (STAT3). STAT3 activation is dependent on the balance between HDAC deacetylation and HAT acetylation [23, 24]. Then, STAT3 acetylation mediates the STAT3–DNMT1 interaction to regulate tumour suppressor gene promoter methylation [25]. STAT3 is hyperacetylated in a number of malignancies [23, 25–30], including ALCLs and malignant T lymphocytes, and acetylated STAT3 was shown to mediate epigenetic tumour suppressor gene (TSG) silencing [31]. HAT and HDAC also regulate the levels of oncoproteins, such as AKT, ABL, Bcr-Abl, and Raf-1, via the acetylation of the mediator (chaperone protein HSP90) in malignant T-cells. Correspondingly, HDACis are capable of depleting pro-growth and pro-survival HSP90 client proteins by targeting the inhibition of HDAC6 [32]. For example, romidepsin plays a key role in pathways involving the chaperone proteins Hsp90 and Hsp70 and the transcription factors C-MYC and p53. Some studies have also investigated whether HDACis are capable of downregulating pro-survival pathway genes such NF-κB [33] and c-FLIP [34] and inhibiting multiple survival signalling pathways, including the PI3K/AKT/mTOR pathway, in peripheral T-cell lymphoma patients (Fig. 2) [35]. In addition, HDACs mediate oncogene activation by the direct intensive histone deacetylation of oncogenes, such as TCRβ, Bcl-xL [20], Bcl2 [36], c-Myc [37], and Notch3 [38], in malignant T lymphocytes. HDAC7 activates the transcription of c-Myc by directly binding to the c-Myc gene, modulating the histone acetylation level of c-Myc gene exon regions and changing the association between RNAP II and the c-Myc gene [39, 40]. Thus, the epigenetic events that occur during T-cell development constitute the molecular basis of T-cell transformation into PTCL. HDAC inhibitor treatment also often mediates the downregulation of these oncogenes in PTCL lymphocytes. HDACs also participate in the transcriptional repression of tumour suppressor genes (TSGs). BIM, which acts as a tumour suppressor in lymphomagenesis, is epigenetically silenced through the following steps: MeCP2 is recruited to methylate CpGs and then binds the SIN3a/histone deacetylase 1/2 (HDAC1/2) corepressor complex to deacetylate histone tails in anaplastic large-cell lymphoma, according to Rocco Piazza’s report [41]. It was confirmed that HDACis are able to restore BIM expression. Both the oncogene and TSG described above are targets of histone acetylation and deacetylation in the pathogenesis of PTCL.

Therefore, histone deacetylase inhibitors (HDACis) can reverse this oncoprotein/oncogene dysregulation and strongly upregulate tumour suppressor gene expression to induce cell death in PTCL by inhibiting the activity of histone deacetylases (HDACs) and restoring the hyperacetylation of histone and non-histone proteins [42]. Histone deacetylase inhibitors elevate the expression level of the proapoptotic protein Bim by promoting the recruitment of E2F1 to the Bim promoter [43]. HDACis also suppress the levels of antiapoptotic Bcl-2 family proteins, including Bcl-XL, Bcl-2, and Mcl-1 [44]. HDAC inhibitors can arrest the lymphoma cell cycle and inhibit cell proliferation by dysregulating the G1-S and S-G2-M checkpoints in the cell cycle [45], inducing lymphoma cell differentiation and promoting the apoptosis of lymphoma cells in PTCL [46]. As reported in the abundant literature, the mechanisms of action of epigenetic drugs have been demonstrated in many haematological malignancy studies. This suggests that histone acetylation targets a wide range of signalling pathways, including pathways involved in DNA repair, the inhibition of angiogenesis, and the generation of reactive oxygen species (ROS). Many of these signalling pathways and oncogenic proteins that play important roles in the pathogenesis of PTCL are listed above, such as the NF-κB, Notch, JAK/STAT3, RHOA, and PI3K/AKT signalling pathways (Fig. 2). Perhaps these are the molecular mechanisms that explain why the use of HDACis in PTCL is breakthrough and compelling. Insights into the molecular mechanisms of epigenetic drugs are lacking in PTCL. HDAC inhibitors work by the following two major mechanisms: increasing promoter histone acetylation and inducing transcription factor acetylation [47]. Consistent with their similar inhibition of the enzyme activity pocket, HDACis seem to have similar toxicity profiles, which include gastrointestinal disturbance, myelosuppression, transient prolongations of QTc interval, nausea, asthenia/fatigue, and infections (all types pooled), although idiosyncratic side effects of particular HDACis have been noted and may relate to differences in chemical structure [48, 49]. Most HDAC inhibitors have similar secondary effects that increase histone acetylation in PBMCs. ABCB1 gene expression was found to be increased in the PBMCs and biopsy samples of peripheral T-cell lymphoma patients [50, 51]. Many studies of other selective HDAC inhibitors are ongoing as stage I or II clinical trials [52]. Romidepsin is a selective class I histone deacetylase inhibitor approved by the FDA in November 2009 for the treatment of recurrent/refractory PTCL. Belinostat, an isohydroxamic acid-type HDAC inhibitor, was approved by the FDA for the treatment of peripheral T-cell lymphoma on July 3, 2014. Chidamide was approved by the CFDA for the treatment of advanced relapsed/refractory PTCL in China as a single agent in 2017. The above drugs can be used to treat PTCL alone or in combination with chemotherapy drugs. Vorinostat (SAHA) was first approved by the FDA in 2006 for the treatment of cutaneous T-cell lymphoma but was also applied to the clinical trial treatment of PTCL.

## Epigenetic drugs in relapsed and refractory PTCL

### Chidamide

Chidamide (HBI-8000 or CS055) is a selective HDACi that contains benzamide compounds. Chidamide exhibited subtype selectivity towards class I HDACs (HDAC1, HDAC2, and HDAC3) and the class IIb HDAC HDAC10 by targeting the catalytic pocket. A study investigated the antitumour effects and the underlying molecular mechanisms of chidamide and showed that chidamide inhibited cell proliferation and arrested cell cycle progression at the G0/G1 phase. In addition, chidamide suppressed the phosphorylation levels of proteins in the AKT/mTOR and MAPK signalling pathways and activated the DDR cell cycle checkpoint pathway (the ATM-Chk2-p53-p21 pathway) in lymphoma [53]. Chidamide is now part of many preclinical and clinical trials in lymphoma patients.

In vitro, the combination of decitabine and chidamide also showed synergistic inhibition in EP300-mutated T-cell lymphomas and KMT2D-mutated tumours [12]. New studies about combined therapy for relapsed/refractory PTCL are ongoing, but more large sample phase III clinical trials are still needed to verify its clinical value to improve the long-term survival of patients with PTCL.

The results from a pivotal multicentre, open-label, phase II study of chidamide in 79 relapsed or refractory peripheral T-cell lymphoma patients showed that chidamide has significant single-agent activity and manageable toxicity in relapsed or refractory PTCL (data in Table 3). In this study, AITL patients responded to chidamide treatment, achieving a higher ORR (50%) and CR/CRu rate (40%) than patients with other subtypes, as well as more durable responses. As such, the China Food and Drug Administration approved chidamide for relapsed or refractory PTCL [54]. A comparison of the treatment options shows that chidamide monotherapy did not significantly prolong the patients’ OS. Nevertheless, the survival benefit from chidamide monotherapy (ORR 29%) was better than that from other second-line treatment regimens such as romidepsin (ORR 25%) and belinostat (ORR 26%). The true usefulness of HDACis might lie in combination with other agents, such as proteasomal inhibitors or kinase inhibitors, which ongoing studies are currently evaluating (Table 5) [55]. According to IPI scores, chidamide can be used to treat recurrent/refractory PTCL alone as well as in combination with chemotherapy drugs. One study enrolled 383 relapsed or refractory PTCL patients to further evaluate the real-world utilization of chidamide (Table 3). In this study, chidamide significantly improved the overall survival rate of patients with angioimmunoblastic T-cell lymphoma (AITL). This large real-world study demonstrates that chidamide monotherapy and combination regimens have favourable efficacy and an acceptable safety profile for refractory and relapsed PTCL patients, especially for PTCL patients with IPI ≥ 2 [56]. The above two studies show that angioimmunoblastic T-cell lymphoma and anaplastic large-cell lymphoma (anaplastic lymphoma kinase-negative) showed better response rates and durable responses than other subtypes [57]. Because of a lack of evident clinical benefit, chidamide is approved only in China. Further investigations are expected to demonstrate that relapsed/refractory PTCL patients could benefit from chidamide.

**Table 3:** Clinical efficiency of epigenetic drugs agents in PTCL

Drug	Patients distribution	Response (ORR, DOR, CR)	Phase
Chidamide [54]	*N* = 79 PTCL-NOS (34%), ALCL (20%), ENKTL: (20%)	ORR:28% Median PFS: 2.1 months OS:21.4 months CR/Cru:14% Median DOR: 9.9 month	II
Chidamide-Combined Chemotherapy [56]	*N* = 127	ORR = 51.18% DCR = 74.02% Median PFS = 152 day	II
Chidamide [56]	*N* = 383 PTCL monotherapy (*n* = 256)	Monotherapy ORR = 39.06% DCR = 64.45%, median (PFS) = 129 days	II
Vorinostat [60] combination with chop	*N* = 14	Median PFS = 79% OS = 82% Median response duration = 29 months	I
Romidepsin [66, 67]	*N* = 130	ORR = 25% CR/Cru = 15% median PFS = 13.4 ~ 29 months. median DOR = 28 months	II
Romidepsin [68]	*N* = 47 PTCL	ORR = 38%,CR = 18% median DOR = 8.9 months	II
Romidepsin plus chop [70]	*N* = 37	ORR = 69% PFS = 41% OS = 71%,	Ib/II
Pralatrexate + Romidepsin [72]	*N* = 29	ORR = 71% (10/14)	II
Romidepsin [73]	*N* = 27 AITL	ORR = 33%	
Belinostat [74]	*N* = 24 PTCL	ORR = 25%, CR = 8%, DOR = 13.6mo	II
Belinostat [75, 76]	*N* = 129	ORR (CR, PR) = 25.8% Median DoR = 13.6 months Median PFS = 1.6 months Median OS = 7.9 months	II
5-Azacytidine [125, 126]	*N* = 12	ORR = 75%, median PFS = 15 months median OS = 21 months	Clinic trial

*AITL* angioimmunoblastic T-cell lymphoma, *PTCL* peripheral T-cell lymphoma, *Median PFS* median progression-free survival, *OS* overall survival, *DOR* duration of response, *Median DOR* median duration of response, *CR* complete response, *DCR* disease control rate, *PTCL-NOS* PTCL not otherwise specified, *ALCL* anaplastic large-cell lymphoma, *ENKTL* extranodal natural killer (NK)/T-cell lymphoma, nasal type

### Vorinostat

Vorinostat (SAHA) was first approved by the FDA in 2006 for the treatment of cutaneous T-cell lymphoma and functions as a pan-HDAC inhibitor that inhibits class I and II HDACs. Vorinostat causes growth arrest as well as caspase-dependent apoptotic cell death and caspase-independent autophagic cell death [58]. In a second phase II trial, vorinostat was used to treat patients with R/R cutaneous T-cell lymphoma (CTCL) and showed an ORR of 30% [59]. Though there are few data on vorinostat in PTCL, one phase I study of vorinostat in combination with a standard CHOP regimen enrolled 14 patients with newly diagnosed peripheral T-cell lymphoma. The phase I study showed the favourable toxicity profile and promising activity of vorinostat plus CHOP (Table 3), but more convincing investigations are needed [60]. However, a vorinostat-containing combination regimen in PTCL was not superior in terms of prolonging overall survival. A phase I/II trial of lenalidomide in combination with vorinostat and dexamethasone for the treatment of relapsed/refractory peripheral T-cell lymphoma (PTCL) indicated poor results [61]. Further animal models and clinical studies are needed to further evaluate the combinatorial effects of this combination.

### Romidepsin

Romidepsin is a typical cyclic depsipeptide class HDACi that has a primarily inhibitory effect on class I HDACs (HDAC1, HDAC2, and HDAC3) and a weak effect on class IIB HDACs (HDAC6). Romidepsin is a more effective single-agent therapy that induces durable responses in relapsed/refractory PTCL patients who have received at least 1 prior systemic therapy, and regardless of age [48, 62–65]. In one pivotal open-label, phase II study of romidepsin treatment in 130 relapsed or refractory peripheral T-cell lymphoma patients after prior systemic therapy, single-agent romidepsin induced complete and durable responses with manageable toxicity in patients with relapsed or refractory PTCL across all major PTCL subtypes, regardless of the number or type of prior therapies [66]. Another update of this study demonstrated an increase in the durable response to romidepsin in a subset of relapsed/refractory PTCL patients. Romidepsin demonstrated long-term tolerability in relapsed/refractory PTCL patients, and the study demonstrated that prolonged romidepsin treatment provided clinical benefits for R/R patients who had achieved at least stable disease. This study is promising for the use of romidepsin as maintenance therapy after induction chemotherapy or after consolidation with high-dose chemotherapy followed by SCT [67]. Phase I and II trials conducted by the National Cancer Institute (NCI) demonstrated the activity of romidepsin in PTCL. In one NCI phase II study (N 47; 45 evaluable for efficacy), clinical activity was demonstrated in patients with recurrent or refractory PTCL [68]. The data described led to the FDA approval of romidepsin in 2011 for the treatment of patients with PTCL following at least one prior therapy. Romidepsin has been recommended by the National Comprehensive Cancer Network (NCCN) as a second-line and subsequent therapy in patients, regardless of the intention to proceed to high-dose therapy or SCT, based on these studies. Many studies are investigating the clinical activity of combination therapies of romidepsin in PTCL [69]. For example, romidepsin plus CHOP is being investigated in a single-arm, phase Ib/II trial of patients with newly diagnosed PTCL (NCT01280526 and NCT01796002), including 37 patients with previously untreated PTCL [70]. Studies of the romidepsin and pralatrexate combination in vitro and in mouse models of T-cell lymphoma have shown that the combination of these agents has synergistic effects, and compared with the same dose of monotherapy, the combined treatment led to a superior tumour reduction [71]. Based on these preclinical data, a phase I/II study of pralatrexate + romidepsin in relapsed/refractory lymphoid malignancies was studied. Twenty-nine patients were enrolled and evaluated for toxicity in this study. The co-administration of pralatrexate and romidepsin was safe and well tolerated. The phase I study of pralatrexate plus romidepsin produced a high response rate in patients with previously treated PTCL. A phase II study in PTCL is needed to further verify the efficacy of this combination. Twenty-three patients were evaluable for response (the results are shown in Table 3) according to the recommended phase I dose [72]. Another investigation of romidepsin showed that it induced durable responses in patients with relapsed or refractory angioimmunoblastic T‐cell lymphoma. This durable response from romidepsin has been supported by a rational basis, which has shown mutations in epigenetic genes in different PTCL subtypes [73]. As a single agent, the efficacy of romidepsin is generally limited, with an ORR of nearly 25% (shown as a combination Table 5). However, when combined with pralatrexate or CHOP, the adverse reactions were not only reduced but also significantly alleviated in PTCL patients, with the ORR reaching 70–80%, which was also consistent with the findings of other reported literature [49] (shown as Table 3).

### Belinostat

Belinostat is a hydroxamic acid-derived pan-HDAC inhibitor that broadly inhibits all zinc-dependent HDAC enzymes, including class I HDACs (HDAC1, HDAC2, and HDAC3), class II HDACs (HDAC6, HDAC9, and HDAC10), and the class IV HDAC HDAC11. Belinostat has been used in relapsed and refractory patients with PTCL (mainly with nodal disease) and CTCL. Belinostat exhibited promising efficacy and a highly favourable safety profile [74]. According to the pivotal phase II BELIEF (CLN-19) study of belinostat in patients with relapsed or refractory peripheral T-cell lymphoma, complete and durable responses could be obtained from belinostat monotherapy in patients with relapsed or refractory PTCL across the major subtypes, irrespective of the number or type of prior therapies. These results have led to the US Food and Drug Administration approval of belinostat for this indication [75, 76]. Many phase I/II trials indicated that belinostat was well tolerated in the treatment of refractory PTCL with manageable toxicity; most of the adverse reactions were mild and transient, and there were no significant blood toxicities [77]. Based on these studies, belinostat seems to be a very attractive candidate for combination therapies due to its favourable safety profile and proven efficacy. A multicentre phase I clinical trial of belinostat combined with the CHOP regimen to treat PTCL patients was completed; in particular, the results showed that the ORR was 86%. This approach might deserve a randomized phase III study in the future comparing this combination therapy with historical standard CHOP therapy [78]. Patients with relapsed/refractory PTCL could benefit from belinostat combination therapy.

### Histone methylation

Histone methylation is another important form of histone modification. The N-terminus of histone methylation is catalysed by histone methyltransferases. H3K4, H3K9, H3K27, H3K36, H3K79, and H3K20 are the main modification sites. Different sites have different modifications, including methylation (me1, me2, or me3), and methylation affects gene transcription levels and leads to the pathogenesis of lymphoma. A study reported that H3K9me-mediated senescence limits the formation of aggressive lymphomas in response to oncogenic Ras [79].

The methylation of lysine 27 on histone 3 (H3K27me) is usually associated with gene repression, but the methylation of lysine 4 on histone 3 (H3K4) is usually associated with gene activation [80, 81]. Under normal circumstances, activating and inhibiting methylation modifications maintain a dynamic equilibrium [82]. EZH2 and MLL2 are a pair of core methyltransferases that regulate the methylation and demethylation of histones, respectively. EZH2 catalyses the trimethylation of lysine 27 of histone H3 (H3K27m3). KMT2D (also known as MLL2) methylates histone H3 at the 4th lysine (H3K4me), and H3K4 methylation at promoters and enhancers is a signature of transcriptional activation (Fig. 1). In addition, SETD2 is a histone lysine methyltransferase responsible for the trimethylation of lysine 36 on histone 3 (H3K36me3), which is an epigenetic marker for active transcription. SETD2 is the most frequently silenced gene in EATL [83], as well as MEITL [84]. Therefore, H3K36 trimethylation is defective in both EATL and MEITL. They also indicated novel roles for SETD2 in T-cell development and lymphomagenesis by modelling the effects of SETD2 loss in vivo. The methylation of histone proteins is involved in the regulation of the cell cycle, lineage commitment, cell senescence, differentiation, and tumourigenesis [85, 86].

EZH2 is a catalytic active component of Polycomb repressive complex 2 (PRC2). The transcriptional inhibition mediated by EZH2 depends on the complete SET domain [87]. Numerous studies on the impacts of EZH2 on malignant B-cell lymphoma have been reported, especially in diffuse large B-cell lymphoma and leukaemia [88]. Few studies on the significance of EZH2 in PTCL have been reported. Enhancer of zeste homologue 2 (EZH2) was found to be widely overexpressed in most T-cell lymphomas, such as NK/T-cell lymphoma (NK/TCL) [89], T-lymphoblastic lymphoma (T-LBL) [90], and adult T-cell leukaemia/lymphoma (ATLL) [91], and was associated with a high proliferation rate and correlated with MYC in ALK + and ALK- ALCL and pSTAT3 expression in ALK + ALCL [92]. In addition, Huilai Zhang et al. systematically reported that high expression of EZH2 in PTCL patients was related to poor prognosis, with high Ki-67 expression being seen in PTCL-NOS [21, 92]. Likewise, targeted sequencing of the main epigenetic modifier genes in a large cohort of Chinese PTCL-NOS patients was performed. The histone modifier genes KMT2C and KMT2D were identified as the most frequently mutated in AITL and PTCL-NOS [93] This study also indicated that the combination of chidamide and decitabine enhances the interaction of KMT2D with the transcription factor PU.1, inactivating the H3K4me-associated signalling pathway MAPK [94], which is constitutively activated in T-cell lymphoma [12, 95, 96].

## DNA methylation

DNA methylation is one of the major epigenetic modifications and plays an important role in regulating gene expression. DNA methylation generally refers to the addition of a methyl group to cytosine in the CpG dinucleotide under the catalysis of a DNA methyltransferase (DNMT) to form 5-methyl cytosine. Hypermethylation of CpG islands in the tumour suppressor gene promoter region leads to gene transcriptional silencing [97], such as the silencing of genes related to cell cycle regulation and tumour suppressor genes (such as p14, p15, and p16) in T-cell lymphoma cells. Treatment of cultured T-cells with the DNA methyltransferase inhibitor 5-aza-2-deoxycytidine reversed p16 gene silencing [98]. STAT3 is involved in the silencing of these TSGs by inducing marked DNA methylation of these gene promoters via the recruitment of epigenetic modifiers, such DNMT1, HDAC1, and MeCP2. Genes in ALCL, such as SHP-1 [99–101], STAT5A [102], and IL2Rγ [103], have previously been recognized to be hypermethylated by NPM-ALK-STAT3 [104]. STAT5A expression is selectively inhibited by the STAT3-mediated DNA methylation of the STAT5A gene promoter region and contributes to ALCL cell growth in cells expressing the oncogenic tyrosine kinase NPM1-ALK (also known as NPM-ALK). Rocco Piazza et al. also demonstrated that NPM/ALK plays a role in initiating the epigenetic silencing process by triggering the methylation of the BIM 5′UTR [41].

DNA methylation is mediated by a family of DNA methyltransferase enzymes, including DNMT1, DNMT3A, and DNMT3B. DNMT1 primarily maintains pre-existing DNA methylation patterns in cell division, whereas DNMT3A and DNMT3B carry out de novo DNA methylation. DNMTs catalyse the transfer of methyl groups to the cytosine nucleotides of CpG island DNA (Fig. 1). Qian Zhang et al. reported that in malignant T-cells, STAT3 induces the expression of DNMT1 [105]. By regulating the expression of DNA methyltransferase 1 (DNMT1) and enhancing the binding of DNMTs to promoters in ALCL, STAT3 regulates TCR complex-related molecules CpG island hypermethylation, expression of TCR complex-related molecules and T-cell identity [106]

DNA structural changes prevent transcription factors from binding, thereby silencing gene expression. The abnormal DNA methylation of tumour-related genes is an early and frequent event in the process of tumourigenesis that contributes to the occurrence and development of lymphoma (Table 1) [107]. Methylcytosine dioxygenase proteins (TET1, TET2, and TET3) convert 5mC to 5-hydroxymethylcytosine (5hmC) (Fig. 1). 5hmC is not maintained by DNMT1, but the change leads to passive demethylation during cell division [108–110].

Recent studies have identified mutations in TET2, IDH2, RHOA, DNMT3A, RHOA, and FYN in PTCL [112]. The mutation frequencies were especially high in peripheral T-cell lymphomas (PTCLs) with features of follicular helper T (Tfh) cells, especially angioimmunoblastic T-cell lymphomas (AITLs) and peripheral T-cell lymphoma not otherwise specified (PTCL-NOS) (Table 1) [7, 111]. Many studies have also shown that TET2, IDH2, and DNMT3A mutations often co-occur in PTCL, and crosstalk between these mutations contributes to the pathogenesis of PTCL [112]. Mutations of TET2 and IDH2 often coexist in PTCL, especially in AITL patients. TET2 and DNMT3A are also frequently mutated in PTCL, which affects the methylation and demethylation of cytosine [14]. Both TET2 and RHOA mutations, in particular, are common in angioimmunoblastic T-cell lymphoma (~ 60–70%) [10, 113].

DNMT3A is the most frequently mutated gene in myeloid and T-cell lymphomas. Staci L. Haney et al. suggested that Dnmt3a is a haploinsufficient tumour suppressor in CD8 + peripheral T-cell lymphoma. Decreased expression of DNMT3A is related to the downregulation of the tumour suppressor p53, which may also contribute to the pathogenesis of CD8 + PTCL [114].

The TET2 gene is considered a tumour suppressor gene that can regulate gene transcription by catalysing DNA demethylation, thus regulating haematopoietic function. Cyril Quivoron et al. investigated Tet2 deficiency in mice and found that TET2 mutations present in lymphoid tumour cells may occur at both the early and later steps of lymphoid development and that the impairment of TET2 function or/and expression predisposes to the development of lymphomagenesis [13]. The highest frequency of TET2 mutations was found in AITL. TET2 mutations were also identified in peripheral T-cell lymphoma not otherwise specified (PTCL-NOS) [115]. Approximately half of all AITL patients carry a loss-of-function mutation in TET2. In addition, loss-of-function mutations of TET2 are quite frequent in lymphomas with follicular helper T-cell-like characteristics [9, 93]. This suggests that TET2 deficiency contributes to the formation of AITL and Tfh-like lymphoma (lymphoma with follicular helper T-cell-like features). Muto H, Sakata-Yanagimoto et al. reported that genetically manipulated murine models with Tet2 knockdown (Tet2gt/gt) develop spontaneous AITL and T-cell lymphoma with Tfh-like features [116].

IDH is a key enzyme in the tricarboxylic acid cycle that can convert isocitric acid into α-ketoglutarate (α-KG). IDH gene mutations generate an enzyme that can convert α-KG to D-2-hydroxyglutarate (D-2-HG) in tumours (Fig. 1). The product of the novel reaction, D-2-HG, interferes with the activity of 2-oxoglutarate-dependent enzymes, perturbing the hypoxic signalling pathway and changing histone methylation and DNA methylation [117]. Thus, the enzymatic function of mutated IDH results in the functional inactivation of TET protein activity [118]. High levels of D-2-HG mediate the inhibition of these α-KG–dependent enzymes, and this is thought to be a possible way that IDH mutations contribute to lymphomagenesis [119].

IDH2 mutations often co-occur with TET2 in AITL [9, 120]. Furthermore, IDH mutations are the second most common genetic abnormality after TET2 mutations in AITL patients. One study analysed the IDH1 and IDH2 genotypes in a set of lymphoma samples that included a large group of PTCLs. IDH2 mutations were identified in approximately 20–45% of angioimmunoblastic T-cell lymphomas [11]. Only the IDH2^R172^ mutation has been identified in AITL. IDH2^R172^ gain-of-function mutations constitute a unique subgroup with distinct TFH-like gene expression signatures in AITL. IDH2^R172^ mutations can induce DNA epigenetic changes and repressive histone hypermethylation in AITL, such as significantly increased levels of histone H3K27me3 and the hypermethylation of promoters, by inhibiting the catalytic activity of the TET2 enzyme [121]. The molecular signature profile heterogeneity in PTCL not only helps us identify and reclassify subtypes but also provides an individualized therapeutic approach that uses agents that specifically target genetic abnormalities or oncogenic pathways found in patients’ tumours [122].

### Treatments related to DNA methylation

Recurrent mutations in TET2, DNMT3A, and/or IDH2 are detected in patients with Tfh-derived PTCL [123] (Table 3). These three genes regulate cytosine methylation and DNA methylation levels. Treatment with hypomethylating agents (HMAs), such as 5-azacytidine and decitabine, shows efficacy in these diseases, and the response rate to HMAs appears to correlate with TET2, IDH1/2, and/or DNMT3A mutations. This suggests that HMAs could have activity against Tfh-derived PTCL. A case report showed that in an AITL patient with TET2 mutation, complete remission was achieved due to the efficacy of 5-azacytidine [124]. A study investigated whether treatment with 5-azacytidine in an AITL patient cohort induces a sustained response and produces lasting complete remission [125]. AITL patients had a higher ORR (75%) than patients with other subtypes (15%), which may be related to the high frequency of TET2 mutations in AITL. The epigenetic changes induced by TET2 mutations offer a rationale for using demethylating agents in TET2-mutated neoplasms [126]. A previous study signified that targeting DNA methylation abnormalities might be an alternative way to treat AITL.

Azacytidine is a cytotoxic cytosine analogue and DNA methyltransferase inhibitor with antineoplastic activity that can reverse the hypermethylation of tumour suppressor gene promoter regions [127]. For example, Kumi Uenogawa et al. demonstrated that azacitidine induces the demethylation of p16^INK4a^, which is implicated as a tumour suppressor gene, and inhibits cell growth in adult T-cell lymphoma [128]. Decitabine (5-aza-2′-deoxycytidine) is a more potent inhibitor of methyltransferases than 5-azacytidine and binds to DNA through a more direct metabolic activation pathway; in addition, it has been shown to have good antineoplastic activity in leukaemia and MDS [129]. Decitabine (5-aza-2′-deoxycytidine) is a hypomethylating agent with a dual mechanism of action: 5-Aza-2′-deoxycytidine is mainly incorporated into DNA, leading to the trapping and depletion of DNA methyltransferases, thus reactivating silenced genes and inducing differentiation at low doses. At high doses, decitabine results in DNA synthesis arrest and cytotoxicity, perturbing cell cycle progression [130]. A study found that 5-aza-Cdr reversed SPARC hypermethylation to restore its biological role as a tumour suppressor in T-NHL cells and inhibited cell proliferation while promoting cell apoptosis [131]. It has been demonstrated that 5-aza-Cdr showed strong antineoplastic activity in anaplastic cell lymphoma (ALCL), causing apoptosis and cell cycle arrest in vitro and in vivo and inhibiting ALK + tumour growth in vitro. Low-dose 5-aza-Cdr treatment of ALCL cell lines and xenograft mouse models also induced the demethylation and re-expression of p16^INK4A^ [132] and the restoration of shp1, which is known to have a tumour suppressor function associated with the downregulation of JAK3/STAT3 signalling in ALK-positive anaplastic large-cell lymphoma [133]. Hypomethylation agents are often combined with other epigenetic modifying drugs in some preclinical studies [134, 135]. Then, molecular basis for this synergistic effect was analysed by evaluating gene-expression and methylation patterns using microarrays. Most of genes were similarly modulated by the combination, but the combination induced an additional transcriptome alteration. Combined therapies have shown synergistic effects in peripheral T-cell lymphoma, resulting in the enhanced induction of lymphoma cell apoptosis [136, 137]. Benigno C. Valdez et al. reported that the combination of a hypomethylating agent, decitabine (DAC), a PARP inhibitor and an HDAC synergistically inhibited cell proliferation and induced apoptosis in human leukaemia and lymphoma cells [138]. A phase I study investigated oral 5-azacytidine (AZA) and romidepsin (ROMI) in patients with PTCL and showed that combination epigenetic modifying drug therapy exhibited marked activity in patients with PTCL [139]. The PTCL responses to the combination proved durable. Moreover, the combination was substantially more active in patients with PTCL than in those with non-T-cell lymphoma.

## Noncoding RNAs regulate peripheral T-cell lymphoma: the role of microRNAs in peripheral T-cell lymphoma

MiRNAs are the most studied noncoding RNAs involved in epigenetic modifications. MicroRNAs (miRNAs) are 18~24-nucleotide single-stranded noncoding RNAs that are highly evolutionarily conserved. MiRNAs function via two main mechanisms: miRNAs can downregulate specific gene products at the posttranscriptional and/or translational level by inducing translational repression or directing mRNA degradation [140]. MiRNAs are involved in the regulation of a series of signal transduction pathways, including cell apoptosis, and play an important role in the pathogenesis of PTCL by regulating the expression of genes at the posttranscriptional level. The roles of specific dysregulated miRNAs that have been reported in lymphoma are listed in Table 4. Distinct microRNA activity signatures are associated with the classification of peripheral T-cell lymphoma subtypes [141].

**Table 4:** List of microRNA (miRNA) expression is dysregulation in ALCL

Subtype of PTCL	Dysregulate microRNA	Biology significance
PTCL-NOS	Overexpressed miR-187 [173]	(1) Associates with high Ki-67 expression, promotes T-lymphoma cell proliferation (2) Relates to tumour progression (3) MiR187 stabilized MYC oncoprotein through Ras-mediated ERK and AKT activation
ALK + ALCL [174]	miR-17 ~ 92 overexpressed miR-135b	(1) Target BIM, identified to have a role in oncogenic ALK signalling in ALCLs (2) Sustain oncogenic properties of STAT3 in T-cell lymphoma [175]
ALK − ALCL	miR-155	expressed more than10-fold higher in ALK − ALCL. [174]
ALCL [176]	Upregulation of miR-135b	(1) miR-135b mediates NPM-ALK–driven oncogenicity, targets GATA3, STAT6, FOXO1 (2) Renders IL-17-producing immunophenotype to anaplastic large-cell lymphoma
ALK + ALCL	miR-16, MiR-29a, miR-96 Downregulation	(1) miR-16 regulate VEGF [177] downregulated (2) MiR-29a inhibiting apoptosis through overexpression of MCL-1 [146] (3) miR-96 a posttranscriptional suppressor, target ALK [178]
ALK( +) ALCL	5 upregulated: 2downregulated miR-155 miR-146a,	(1) miR-17 ~ 92 is an important downstream effector of ALK oncogenic pathway[179]
ALK(−) ALCL	4 Upregulated: 7 Downregulated:	(1) The signature of a series of 11 miRNAs distinguishes ALK (−) ALCL from other PTCLs. (2) Classification of peripheral T-cell lymphoma subtypes [179, 180]
ALCL	miR-101 was downregulated in all ALCL cell lines,	(2) miR-101 in the regulation of mTOR pathway [174]
AITL PTCL-NOS	BCL6 and a specific set of miRNAs	Mutual regulation between BCL6 and a specific set of miRNAs controls the TFH phenotype in peripheral T-cell lymphoma [181]
Peripheral T-cell lymphoma not otherwise specified	Expression levels of miRNA	(1) Discriminating PTCL NOS from activated CD4 + and CD8 + T-lymphocytes, such as AITL and ALCL (2) miR-132-3p is also an important modulator of the PTCL NOS transcriptome [182]
PTCL NOS	13 miRNAs upregulated and 7 miRNAs downregulated	the potential to be used as biomarkers for the identification of patients with PTCL NOS [183]
Angioimmunoblastic T-cell lymphoma. In AITL,	Upregulated: miR-146a, miR-193b miR-34a downregulated miR-30b	Both miR146-a and miR-30b contribute to the pathogenesis of AITL [184]
ALK( +) ALCL	miR-21 Downregulated	Targets DNMT1 mRNA [103]

The inverse correlation of promoter DNA methylation and expression of mature miRNAs has been most reported [142]. Some tumour suppressor miRNA genes might be silenced by the DNA hypermethylation and closed chromatin structure around their promoter regions in PTCL. MiRNA epigenetic inactivation in NPM–ALK( +) ALCL is mediated by upregulating and recruiting DNMT1 to the promoter of miRNA. Only a few studies regarding miRNA expression in PTCLs have been reported so far, all focusing particularly on ALCLs. Olaf Merkel showed that DNA methylation of the promoter of the miR-155 host gene inhibited the expression of miR-155 [143, 144]. Tumour-suppressive miRNAs are often methylated (inactivated) in T-cell lymphomas, leading to the upregulation of target oncogenes [145]. As tumour-suppressive miRNAs, the expression of miR-29a [146], miR129-2, Hsa-miR-203, miR-195, miR-497, miR125b, and miR150 was suppressed by promoter hypermethylation in ALK-positive ALCL [146]. MiR129 frequently undergoes methylated inactivation in T-cell lymphomas [147] and often collaborates with MiR124-1 and MiR203 methylation in T-cell lymphomagenesis [145]. MicroRNA methylation was associated with the concomitant methylation of other miRNAs in PTCL. The Hsa-miR-203 promoter has been reported to be frequently methylated in T-cell lymphoma, [148] with the concomitant methylation of hsa-miR-34a, −124a and −196b [149].

MiRNAs affect the expression of epigenetic modifying genes, and the downregulation of miR-101 and miR-128a may be responsible for increased EZH2 expression in T-cell lymphoma oncogenesis [150]. In an NHL case–control population, Alan Fu et al. demonstrated that reduced levels of mature miR-618 may lead to the deregulation of the targeted genes HAT1/HDAC3. The inactivation of miR-497 in human NPM-ALK ( +) anaplastic large-cell lymphoma cells promotes cell growth by dysregulating targeted CDK6, E2F3, and CCNE1, the three regulators of the G1 phase of the cell cycle [151]. These findings suggest that some miRNA genes are controlled by epigenetic alterations in their promoter regions and can be activated by inhibitors of DNA methylation and HDAC.

## Conclusion and perspective

The treatment of PTCL remains clinically difficult. The response rate to first-line treatment for PTCL is low, and the current 5-year overall survival is only 10–30%. As epigenetic data in lymphoma have been continually reported in recent years, PTCL outcomes have improved, and histone deacetylase inhibitors can achieve a high rate of response and result in durable remission. As shown in Table 3, the ORR of belinostat, romidepsin, and chidamide in RR PTCL was similar and slightly higher in the single drug treatment group than in the CHOP group. However, when HDACI was combined with chemotherapy, the ORR was significantly improved, reaching as high as 59–79%. The associated toxicities of HDACis, such as cardiotoxicity and haematological and gastrointestinal effects, are largely controllable. Therefore, novel epigenetic combination treatments based on biological pathophysiology, preclinical data, and clinical efficacy are needed to challenge front-line conventional chemotherapy in the future. 5-Azacytidine and romidepsin combination regimens still suit those patients scheduled for SCT, as reported in a phase I study [139]. The combination of epigenetic drugs and immune checkpoint inhibitors such as anti-PD1 mAb and lenalidomide are expected to have a good therapeutic effect. Ongoing studies are testing these new agents in combination with chemotherapy in the front-line setting [55] (Table 5). However, the genomic instability and genotoxicity induced by HDACis remain to be elucidated [152]. The diagnosis of PTCL was mainly based on clinical presentation, morphological features, and immunophenotypes in the past. Recent advances in genome sequencing and gene expression profiling have provided new insights into the pathogenesis and molecular biology of PTCL. In particular, some recurrent epigenetic modifying gene mutations, miRNA expression signatures and histone modifications can serve as biomarkers for the diagnosis, management, and prognosis of PTCL [7]. The crosstalk between epigenetic modifier mutations contributes to PTCL lymphomagenesis and sensitizes lymphoma cells to epigenetic treatment.

**Table 5:** Novel HDACi combination therapies under investigation for relapsed/refractory peripheral T-cell lymphomas (Refer to Enrica Marchi et al.) [55]

Combination trials	Mechanism of action	Phase	ClinicalTrial.gov ID
Chidamide + CHOP	Anthracycline-containing regimens	I	NCT02809573
Chidamide + Cyclophosphamide + Thalidomide	HDAC inhibitor + Immunomodulatory drugs	II	NCT02879526
Romidepsin + CHOEP	Anthracycline-containing regimens	I/II	NCT02223208
Romidepsin + CHOP	Anthracycline-containing regimens	III	NCT01796002
Romidepsin + ICE	Anthracycline-containing regimens	I	NCT01590732
Romidepsin + Lenalidomide	HDAC inhibitor + Immunomodulatory drug	II I/II	NCT02232516 NCT01742793
Belinostat + Carfilzomib	HDAC inhibitor + proteasome inhibitor	I	NCT02142530
Pralatrexate + Romidepsin	HDAC inhibitor + antifolate	I/II	NCT01947140
Romidepsin + 5-Azacitadine	hypomethylation agent + HDAC inhibitor	I/II	NCT01998035

Differential miRNA expression also has the potential to be used as a biomarker for the subtype identification and diagnosis of PTCL. DNA methylation of the promoter often leads to miRNA deregulation, which has been demonstrated to play a role in the pathogenesis of ALK( +) ALCL and PTCL-NOS [153]. The challenge in the future will be to fully understand the epigenomes and epigenetic mutations in peripheral T-cell lymphoma to find more reliable early diagnostic markers and clearer therapeutic targets to provide new directions and therapeutic strategies for the research and treatment of relapsed peripheral T-cell lymphoma.

## Abbreviations

AITL: Angioimmunoblastic T-cell lymphoma
ALK + ALCL: ALK-positive anaplastic large-cell lymphoma
ALK-ALCL: ALK-negative anaplastic large-cell lymphoma
CHOP: Cyclophosphamide + doxorubicin + vincristine + prednisolone
EATL: Enteropathy-associated T-cell lymphoma
MEITL: Monomorphic epitheliotropic intestinal T-cell lymphoma
JAK: Janus kinase
PI3K: Phosphoinositide 3-kinase
AKT: Protein kinase B
PTCL: Peripheral T-cell lymphoma
PTCL, NOS: Peripheral T-cell lymphoma not otherwise specified
PTCL-TFH: Nodal peripheral T-cell lymphoma with TFH phenotype
FTCL: Follicular T-cell lymphoma
NHL: Non-Hodgkin lymphoma
TET: Ten eleven translocation protein
2HG: 2-Hydroxyglutarate
IDH2: Isocitrate dehydrogenase
2,5HmC: 5-Hydroxymethylcytosine
EZH2: Enhancer of zeste 2
PFS: Progression-free survival
OS: Overall survival
DOR: Duration of response
DOR: Duration of response
CR: Complete response
DCR: Disease control rate
STAT: Signal transducer and activator of transcription
